# Multispectral Emissions of Lanthanide-Doped Gadolinium Oxide Nanophosphors for Cathodoluminescence and Near-Infrared Upconversion/Downconversion Imaging

**DOI:** 10.3390/nano6090163

**Published:** 2016-09-06

**Authors:** Doan Thi Kim Dung, Shoichiro Fukushima, Taichi Furukawa, Hirohiko Niioka, Takumi Sannomiya, Kaori Kobayashi, Hiroshi Yukawa, Yoshinobu Baba, Mamoru Hashimoto, Jun Miyake

**Affiliations:** 1 Graduate School of Engineering Science, Osaka University, 1-3 Machikaneyama, Toyonaka, Osaka 560-8531, Japan; dkdung@bpe.es.osaka-u.ac.jp (D.T.K.D.); syou_fukushima@sml.me.es.osaka-u.ac.jp (S.F.); mamoru@me.es.osaka-u.ac.jp (M.H.); jun_miyake@bpe.es.osaka-u.ac.jp (J.M.); 2 Institute for NanoScience Design, Osaka University, 1-3 Machikaneyama, Toyonaka, Osaka 560-8531, Japan; furukawa@insd.osaka-u.ac.jp; 3 Department of Innovative and Engineered Materials, Tokyo Institute of Technology, 4259 Nagatsuta, Yokohama, Kanagawa 226-8503, Japan; sannomiya.t.aa@m.titech.ac.jp; 4 Graduate School of Engineering, Nagoya University, Furo-cho, Chikusa-ku, Nagoya 464-8603, Japan; kobayashi.kaori@d.mbox.nagoya-u.ac.jp (K.K.); h.yukawa@nanobio.nagoya-u.ac.jp (H.Y.); babaymtt@apchem.nagoya-u.ac.jp (Y.B.); 5 ImPACT Research Center for Advanced Nanobiodevices, Furo-cho, Chikusa-ku, Nagoya 464-8603, Japan; 6 Health Research Institute, National Institute of Advanced Industrial Science and Technology (AIST), 2217-14, Hayashi-cho, Takamatsu 761-0395, Japan

**Keywords:** bio-imaging, multimodal imaging, cathodoluminescence, near-infrared imaging, nanophosphors

## Abstract

Comprehensive imaging of a biological individual can be achieved by utilizing the variation in spatial resolution, the scale of cathodoluminescence (CL), and near-infrared (NIR), as favored by imaging probe Gd_2_O_3_ co-doped lanthanide nanophosphors (NPPs). A series of Gd_2_O_3_:Ln^3+^/Yb^3+^ (Ln^3+^: Tm^3+^, Ho^3+^, Er^3+^) NPPs with multispectral emission are prepared by the sol-gel method. The NPPs show a wide range of emissions spanning from the visible to the NIR region under 980 nm excitation. The dependence of the upconverting (UC)/downconverting (DC) emission intensity on the dopant ratio is investigated. The optimum ratios of dopants obtained for emissions in the NIR regions at 810 nm, 1200 nm, and 1530 nm are applied to produce nanoparticles by the homogeneous precipitation (HP) method. The nanoparticles produced from the HP method are used to investigate the dual NIR and CL imaging modalities. The results indicate the possibility of using Gd_2_O_3_ co-doped Ln^3+^/Yb^3+^ (Ln^3+^: Tm^3+^, Ho^3+^, Er^3+^) in correlation with NIR and CL imaging. The use of Gd_2_O_3_ promises an extension of the object dimension to the whole-body level by employing magnetic resonance imaging (MRI).

## 1. Introduction

In recent years, multimodal imaging has attracted tremendous attention among researchers in varied fields [1,2,3,4,5,6,7,8]. The principal merit of multimodalities is the capability of each modality to synergistically and significantly compensate for the limitations of the others. Multimodal correlative imaging enables the comprehensive study of biological entities. In particular, it yields accurate visions of biological processes, sub-cellular molecules, and cellular and tissue architecture/morphology in synergy with the anatomical details of dynamic processes or morphological changes [3,4,9]. The most widespread combination to visualize sub-cellular components arises from the co-registration of electron microscopy (EM) and light microscopy (LM). At a cellular level, light microscopy (LM) is a versatile tool that is capable of obtaining specific and functional information with high sensitivity. An optical image from conventional microscopy is restricted to identifying ultrastructural details of biological components and cellular molecules, whereas with electron microscopy (EM) an extreme near-atomic-level resolution has been accomplished. Correlative light and electron microscopy (CLEM) is by far the most interest paradigm in identifying specific structures of sub-cellular life [10,11]. However, substantial challenges in discriminating multiple molecules due to the monochromic images limit the efficiency of CLEM. Moreover, imaging at deep regions by using conventional light microscopy is hindered due to light absorption and scattering by tissue components [12,13,14]. To overcome this challenge, our group has proposed a combination of cathodoluminescence (CL) and near-infrared (NIR) imaging [15]. This model initiated the correlation of CL electron microscopy, where the high resolution of electron microscopes is associated with spectral discrimination, and NIR, where the light in the near-infrared region can extend the penetration into deep regions. The correlation can provide a visual observation of multiple components in varied tissue-cell-molecular scales.

The combination of CL and NIR is constructed by the integration of two imaging techniques along with the design of favorable multimodal probes. In our previous results, Tm^3+^/Yb^3+^ co-doped yttrium oxide nanophosphors were used [15]. CL emissions of Y_2_O_3_ doped with lanthanides (Ln) are attributed to the transitions of electrons within the electronic energy levels that are well shielded from the host lattice. Therefore, emissions from Ln^3+^ sites are narrow and well-defined [16]. Furthermore, lanthanide (Ln^3+^/Yb^3+^) co-doped nanophosphors (NPPs) are tunable for exhibiting a multispectral luminescence spanning from the ultraviolet (UV) to NIR regions under 980 nm excitation or electron irradiation [16,17,18,19,20,21,22].

Cathodoluminescence involves the emission of photons of characteristic wavelengths when the surfaces of nanoparticles are bombarded with accelerated electrons. This phenomenon is assigned to the electron transition from the valence band to the conduction band of materials which favor impure atoms or dopants [23,24]. In lanthanide-doped nanophosphors, the emission wavelengths are characterized by the dopant elements. The most common dopant for CL with lanthanide phosphors is Eu^3+^ with red light. The multicolor correlation of CL and LM with NPPs has been conducted by using Eu^3+^, Tb^3+^, and Tm^3+^ dopants [16,25]. An addition of Yb^3+^ facilitated the correlation of CL and NIR of Y_2_O_3_ doped with Tm^3+^ [15].

Regarding NIR imaging, upconverting (UC) and downconverting (DC) emissions are of great interest as the light in this region can penetrate into biological tissues more efficiently than visible light [12]. The penetration depth of light through tissue is determined according to the interactions between photons and the media, particularly the scattering and absorption of photons [13,14]. Light scattering in tissue is exponentially attenuated according to Rayleigh’s law, to the fourth power of the wavelength (I~λ^−4^) as the wavelength increases [26], whereas the light absorption coefficient by hemoglobin diminishes significantly in the NIR region. Notwithstanding that, the contributions to tissue absorption by water become significant as the wavelength increases higher than 950 nm; there are clearer regions where tissue components have their lowest absorption [12,13,26,27,28]. The tissue-optical-transparent windows exist at 650–950 nm, 1000–1350 nm, and 1600–1870 nm, named the NIR-I, NIR-II, and NIR-III biological windows, respectively [12,13,28,29]. The first window, where the light has a wavelength from 650 to 950 nm, is still in the active region of auto-fluorescence [12]. However, the transmittance of light through the skin is relatively high around 810 nm [30]. The light in the ranges of 1000–1350 nm and 1600–1870 nm for the second window and the third window, respectively, provides deep penetration for imaging [12,13,14]. An increase of the signal-to-noise ratio by using quantum dot (QDs) fluorophores, which emit at 1320 nm in the second window (instead of 850 nm), was demonstrated [12]. The penetration of the light of wavelengths longer than 1500 nm through muscle tissues was also recorded [31].

Lanthanide-doped nanophosphors (NPPs) are tunable for exhibiting multispectral luminescence spanning from the UV to the NIR regions under 980 nm excitation or electron irradiation [16,17,18,19,20,21,22,32,33]. To investigate the spectral distinction of lanthanide-doped NPPs for correlating CL and NIR, Tm^3+^/Yb^3+^, Ho^3+^/Yb^3+^, and Er^3+^/Yb^3+^ ions were selected for the activator/sensitizer association in this work. An appropriate activator/sensitizer dopant concentration is one of the most important factors for getting the highest emission intensity at a distinct wavelength for NIR, as it is well known that the luminescence intensity depends on the sensitizer/activator concentration in NIR emissions [34,35,36]. The concentration quenching varies among the sensitizer/activator combinations. This ratio also affects the emission intensity in CL.

In order to exploit the multispectral emission of Tm^3+^/Yb^3+^, Ho^3+^/Yb^3+^, and Er^3+^/Yb^3^^+^ for NIR and CL, the UC/DC emission intensity and dopant ratio dependence are first optimized quantitatively by using Gd_2_O_3_ powder synthesized by the sol-gel method. These ratios are used to produce nanoparticles by the homogeneous precipitation (HP) method. The CL emissions from these particles are then investigated. The imaging by NIR and CL are also conducted to obtain emissive images of nanoparticles. The use of gadolinium oxide (Gd_2_O_3_) as a host material instead of Y_2_O_3_ is interesting because it has intrinsic magnetic properties, which permits the expansion of potential applications using magnetic resonance imaging (MRI) [37,38,39]. Gadolinium ions possess seven unpaired electrons, which accelerate the proton relaxation in MRI, a result of which is T_1_ contrast enhancement. It allows the addition of imaging modalities for further applications. 

## 2. Experimental Details

### 2.1. Materials

Gadolinium nitrate hexahydrate Gd(NO_3_)_3_·6H_2_O 99.99% in purity, erbium nitrate hexahydrate Er(NO_3_)_3_·6H_2_O 99.9% in purity, and Triton-X 100 were purchased from Sigma-Aldrich (St. Louis, MO, USA). Holmium nitrate hexahydrate Ho(NO_3_)_3_·6H_2_O 99.9% in purity, thulium nitrate hexahydrate Tm(NO_3_)_3_·6H_2_O 99.9% in purity, and ytterbium nitrate pentahydrate Yb(NO_3_)_3_·5H_2_O 99.9% were purchased from Kojundo Chemical Laboratory Co., Ltd. (Saitama, Japan). Urea CO(NH_2_)_2_ and l-Glutamic acid were purchased from Wako Ltd. (Osaka, Japan). All of the chemicals were used without any further purification.

Dulbecco’s Modified Eagle Medium (DMEM) was purchased from Sigma-Aldrich. A 10% (vol/vol) fetal bovine serum from Nichirei Bioscience Inc. (Tokyo, Japan), 1% (vol/vol) penicillin streptomycin from Wako Ltd., and 25 µg/mL amphotericin B from Sigma-Aldrich were added to the DMEM. Phosphate buffered saline (PBS (−)) and 4% paraformaldehyde were purchased from Wako Ltd.

### 2.2. Synthesis of Nanophosphors

A synthesis strategy is a critical factor to suppress luminescence quenching regardless of other factors such as the host matrix or dopant ratios. The sol-gel method is a productive, facile, and low-cost method for the preparation of large-scale multi-component materials with different shapes [40,41,42,43]. Moreover, this method can control the concentration of composition precisely to the atomic level, where the components distribute homogeneously on an atomic scale. It is very useful to produce large amounts of materials and the variation of composition concentration is very small. Therefore, for the experiments of determining the favorable dopant ratios in characteristic wavelengths of Gd_2_O_3_ doped with lanthanides, the sol-gel method was employed to produce the materials. The dopant ratios were investigated for the emissions from the visible to NIR region. The sensitizer/activator ratios were sequentially variable from 0.1 to 5 mol %. This work can serve as a reference for the dopant ratios, which provide the highest UC/DC emission intensity in further experiments using Gd_2_O_3_ doped with lanthanide nanophosphors. On the other hand, the HP method is commonly used to produce a high yield of dispersed lanthanide-doped nanophosphors. The HP method is incomparable to the sol-gel method in terms of production yield; however, its benefit is in the production of monodisperse nanoparticles, which facilitates their applications in bioimaging. The nanoparticles from the HP method are used in CL and NIR bioimaging.

### 2.3. Sol-Gel Method

This method produces oxide nanocrystal networks from precursors of metal inorganic salts; in this work Ln^3+^ nitrates were used, and a chelating agent which favors hydrolysis and polycondensation reactions [41]. This process can produce a multi-component oxide material with a well-controlled stoichiometry, high purity, and chemical homogeneity. Through this process, the microstructure and composition at the atomic level can be controlled, and the optical properties are able to be tailored [42,43].

Ln^3+^ + 3 C_5_H_9_NO_4_ → Ln (C_5_H_8_NO_4_)_3_ (1)

**Scheme 1:**
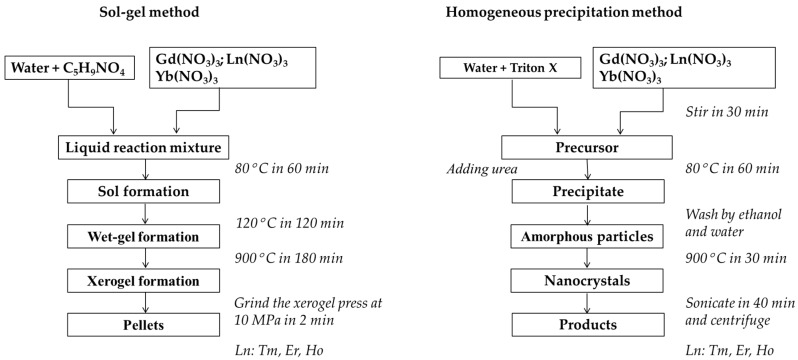
The synthesis routes for preparation of Gd_2_O_3_ doped with lanthanides.

The precursor contains 15 mL of 120 mM aqueous solutions of gadolinium and lanthanide nitrate salts. The composition was sequentially varied for the stoichiometry Gd: x(Ln): y(Yb), mol %; (Ln: Tm, Ho, Er), where (x, y = 0.1, 0.2, 0.5, 1, 2, 5). The precursors were mixed with 256 mg L-Glutamic acid and stirred at 80 °C for 60 min. The mixture then formed a compound as shown in Equation (1). A viscous solution appeared after increasing the temperature to 120 °C within 2 h and finally transformed into a wet gel. This gel transformed to the oxide phase after a heating treatment at 900 °C for 3 h. The porous networks of nanocrystals were obtained after the calcination. The synthesis process is described in Scheme 1. The products were grinded and pressed into pellets at 10 MPa for measurement of UC/DC emissions.

### 2.4. Homogeneous Precipitation (HP) Method

This method utilizes the thermal decomposition of urea to control the release of OH^−^ constituents and consequently controls the nucleation and growth of the heterogeneous system Ln_2_O_3_–H_2_O–CO_2_ [44].

(2) $${\mathrm{Gd}}^{3+}+{\mathrm{OH}}^{–}+{\mathrm{CO}}_{3}^{2–}+1.5\;H_{2}O\to \mathrm{Gd}\;(\mathrm{OH}){\mathrm{CO}}_{3}\cdot 1.5\;H_{2}O↓$$

By adding surfactants, the size and shape of the nanoparticles are able to be controlled. Triton−X 100 is a non-ionic and harsh surfactant. It was used to avoid aggregation and assist the dispersion of the nanoparticles. The schematic illustration of the synthesis of Gd_2_O_3_ co-doped Tm^3+^, Er^3+^, Ho^3+^/Yb^3+^ is shown in Scheme 1.

80 mL of 5 mM aqueous gadolinium nitrate and other lanthanide nitrate salts of Tm/Yb, Ho/Yb, or Er/Yb were stirred at room temperature with an appropriate amount of Triton-X 100 for 30 min. Then 24 g urea was added and the temperature was increased to 80 °C and held for 60 min. The final slurry was consequently centrifuged at 12,000 rpm, and repeated several times to remove the remaining surfactants. The nanoparticles were collected and dried at 80 °C before calcination at 900 °C for 30 min. Final products were sonicated for about 40 min for further analysis and imaging.

### 2.5. Characterization

Transmittance electron microscopy (TEM): Carbon grids were dipped in an ethanol solution containing NPPs, and dried at room temperature. The size and morphology of the NPPs were determined using a transmission electron microscope (Hitachi Model H-7650).

Upconverting/downconverting spectra: The UC and DC luminescence were recorded at 298 K using an ultraviolet-visible spectrometer (QE 65 Pro, Ocean Optics) and a near-infrared spectrometer (NIRQuest512, Ocean Optics), respectively. The samples were excited by a 980 nm continuous-wave (CW) diode laser (IRM980TR−500, Laser Century). A 950 nm short-pass filter (FF01−950/SP−25, Semrock) and a 980 nm long-pass filter (RazorEdge LongPass 980, Semrock) were inserted in a dual 90° flip mount to categorize the luminescence before transferring it to the spectrometer. The short-pass filter was used to obtain the UC emission and the long-pass filter was used for the DC emission. The detailed information about the optical setup is presented in Figure S1 of the Electronic Supplementary Materials.

NIR imaging: HeLa cells (Health Science Research Resources Bank, Osaka, Japan) were cultured on a glass bottom dish in DMEM with 10% fetal bovine serum and 1% penicillin streptomycin at 37 °C and in 5% CO_2_. Gd_2_O_3_ co-doped Tm^3+^/Yb^3+^, Ho^3+^/Yb^3+^, and Er^3+^/Yb^3+^ nanoparticles obtained from the HP method were sterilized by exposure to UV light for 24 h and re-dispersed in DMEM. The nanoparticles in the medium were then added into the cellular dishes and incubated for 24 h. The cell uptake of nanoparticles is enabled via the endocytosis pathway. The samples were washed several times with PBS (−) and then fixed by 4% paraformaldehyde for 20 min. The observation was conducted by using a laser scanning microscope (C1, Nikon) integrated with a self-constructed optical setup. The integrated setup allows for obtaining the luminescence using a 980 nm laser source. The information about the optical system is explained in detail in the Electronic Supplementary Materials Figure S2.

Cathodoluminescence: CL images from three kinds of Gd_2_O_3_ nanoparticles were obtained by using a self-constructed CL system integrated with a STEM system (JEM–2100F). The emitted photons from the samples under the electron beam irradiation were collected by using a parabolic mirror. The samples were mounted on the focus of the parabolic mirror so that the emissions above and below the samples can be reflected onto the inner surface of the mirror and form parallel rays. The parallel rays exit from the STEM instrument through a quartz window, and were then focused in front of a slit of the spectrometer (Andor, SR163, *f* = 163 mm, *F*/3.6). The CL signal was recorded by an Electron Multiplying Charged Couple Device (EM-CCD) (Andor, DU920P–BU). Even though the emission spectra from the whole observation area was recorded, the CL images of nanoparticles were reconstructed at specific wavelengths. Where the background emission originated from the transition radiation of the host material was fitted by a quadratic function and was subtracted from the observed spectrum because the spectral bandwidth of the host materials was much wider than that of the dopant elements. The CL spectra were obtained from the large aggregated nanoparticles. The background in the whole spectra region was also fitted by a polynomial function and subtracted to obtain the emissions from the nanoparticles. More detailed information about the STEM-CL imaging can be found in Figure S3 of the Electronic Supplementary Materials.

## 3. Results and Discussion

### 3.1. Morphology of NPPs

#### 3.1.1. The Sol-Gel Method

**Figure 1:**
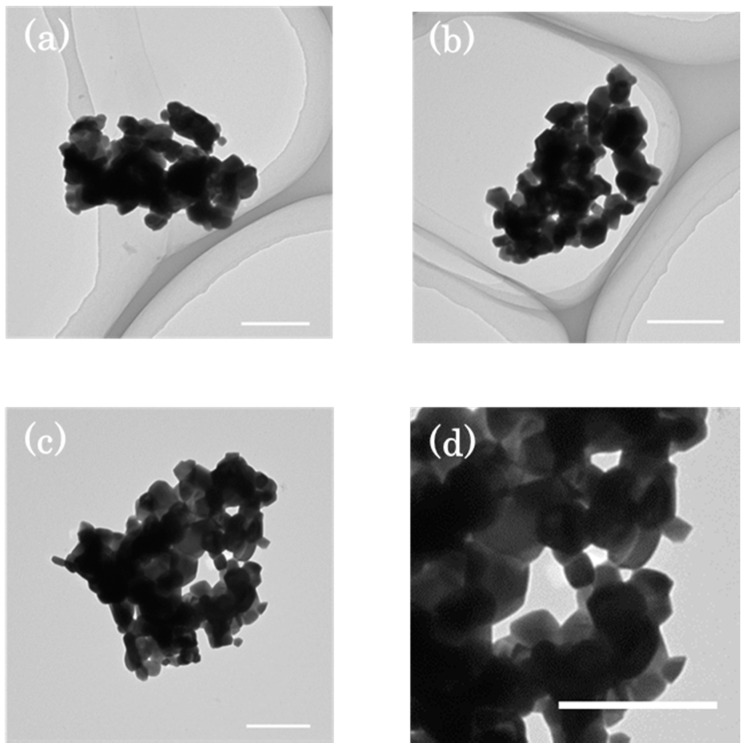
(**a**–**c**) TEM images of Gd_2_O_3_ powder from the sol-gel method: Gd_2_O_3_ co-doped (**a**) Tm 0.1 mol %/Yb 2 mol %; (**b**) Ho 1 mol %/Yb 2 mol %; and (**c**) Er 1 mol %/Yb 2 mol %. (**d**) is an enlarged of Figure 1c. The scale bars indicate 200 nm.

The morphologies of the Gd_2_O_3_ powder produced by the sol-gel method are shown in Figure 1a–d. Figure 1a is a TEM image of Gd_2_O_3_ co-doped Tm 0.1 mol %/Yb 2 mol %, Figure 1b is an image of Gd_2_O_3_ co-doped Ho 1 mol %/Yb 2 mol %, and Figure 1c is an image of Gd_2_O_3_ co-doped Er 1 mol %/Yb 2 mol %. As seen in the figure, the xerogel structure of NPPs made from the sol-gel method exhibit a particulate morphology. These particles form an interconnected network of nanocrystals. During calcination, the macropore shrinkage within the network was induced by the densification of the materials. In Figure 1d, an enlarged image of Figure 1c, particles in the crystal network are highly aggregated. The particle boundary is fully fused with the other particles. This is the most difficult issue with applying sol-gel products in CL and NIR bio-imaging, where the particle size and dispersion contribute significantly to the image resolution. Large aggregates emit strong luminescence; however, the bulky size of the powder is larger than the molecular target size. Therefore, the determination of the molecular position is impossible. Nevertheless, the superiority in composition control at the atomic level allows this method to provide a high yield of homogeneous materials with precise components. This is helpful for evaluating the dependence of the dopant ratio versus the NIR luminescence intensity.

#### 3.1.2. Homogeneous Precipitation (HP) Method

**Figure 2:**
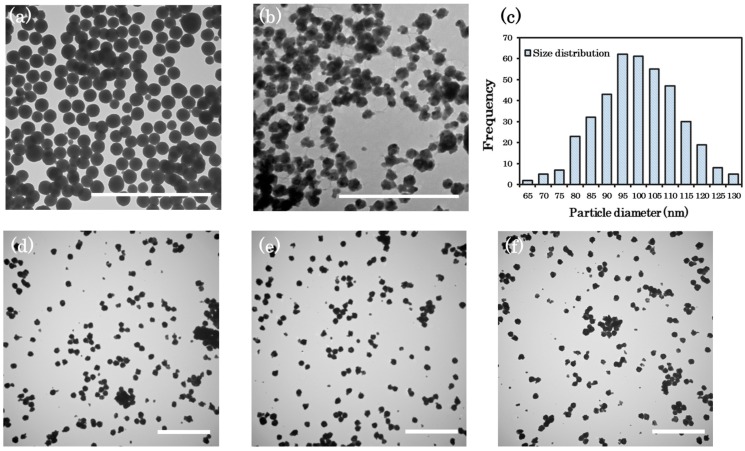
(**a**) and (**b**) show the TEM images of Gd_2_O_3_ co-doped Ln^3+^/Yb^3+^ nanoparticles generally produced from the HP method before and after calcination, respectively. The size distribution of nanoparticles pre-calcination synthesized from the HP method is shown in (**c**); The calcined nanoparticles of Gd_2_O_3_ co-doped (**d**) Tm 0.1 mol %/Yb 2 mol %; (**e**) Ho 1 mol %/Yb 2 mol %; and (**f**) Er 1 mol %/Yb 2 mol % underwent a sonication to demolish the aggregates and obtain the dispersed particles. The scale bars indicate 1 µm.

The particles from the HP method have a spherical shape as shown in the TEM images in Figure 2. The TEM images of as-synthesized particles and post-calcinated nanoparticles are shown in Figure 2a,b, respectively. The size distribution in Figure 2c shows a particle diameter of around 103.6 ± 14.16 nm. The distribution was estimated by analyzing the particle diameters using TEM images of 400 nanoparticles. The software ImageJ, and in particular the ‘analyze particles’ function (National Institutes of Health), was used for this work. The nanoparticles underwent a heating process up to 900 °C to eliminate the remaining carbonate or hydroxide phases that cause the quenching of luminescence [41,42], which transformed the particles into nanocrystals of oxide phases. Sintering—a consequence of calcination—was unavoidable because the mobility of the atoms was triggered at the high temperature, inducing a diffused flux of materials. The monodispersed particles, as synthesized, transformed into large aggregates. However, these aggregates are loose and can be demolished by further sonication, as shown in Figure 2d–f. The particles after calcination of Gd_2_O_3_ co-doped Tm 0.1 mol %/Yb 2 mol %, Ho 1 mol %/Yb 2 mol %, and Er 1 mol %/Yb 2 mol % are shown in Figure 2d–f, respectively.

### 3.2. Upconverting/Downconverting Luminescence Properties

#### 3.2.1. Mechanism of UC/DC Emissions

**Scheme 2:**
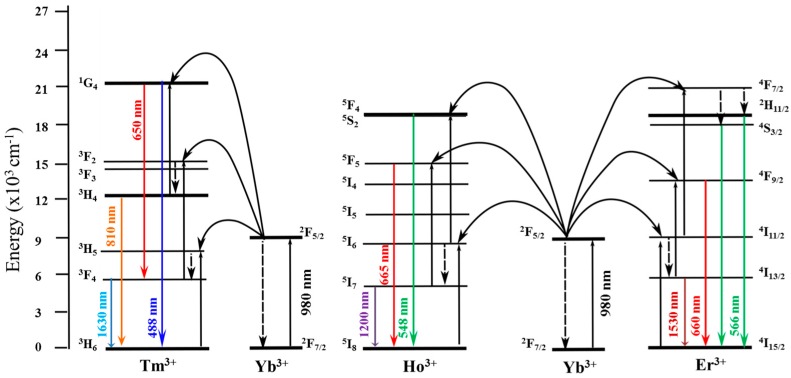
Energy level diagram of possible emitting levels, as well as radiative and non-radiative pathways [30,31,32,33]. Excitation and emission processes are represented by upward and downward solid arrows, respectively. The curved and dashed arrows denote energy transfer and non-radiative relaxation processes, respectively.

An analysis of the possible emitting transitions of Gd_2_O_3_:Ln^3+^/Yb^3+^ (Ln^3+^: Tm^3+^, Ho^3+^, Er^3+^) are depicted in Scheme 2. The up/downconverting emissions under 980 nm excitation take place thanks to the Yb^3+^ ions. Yb^3+^ has only the excited level ^2^F_5/2_ in the 4f shell, which coincides with 980 nm; hence, the absorption cross-section at this wavelength is much larger than that of the Tm^3+^, Ho^3+^, and Er^3+^ ions. According to the energy diagram, the Yb^3+^ ion absorbs the photon from the 980 nm laser, and then transfers its energy to other activators (Tm^3+^, Ho^3+^, Er^3+^), which results in an excitation to a long-lived high-energy level [45]. The UC/DC luminescence can be obtained due to a sequential multi-photon energy transfer process from the excited ^2^F_5/2_ of Yb^3+^ [45].

The blue and red emissions of Tm^3+^ originate from the energy populations at the ^1^G_4_ level of Tm^3+^. The multiphonon relaxation of the ^3^H_5_ state was induced after energy transferred from the ^2^F_5/2_ level of Yb^3+^, resulting in a population at the ^3^F_4_ level in Tm^3+^. The second and the third excitations are ascribed to energy transfers from Yb^3+^ to the ^3^F_2_ and then ^1^G_4_ states of Tm^3+^, respectively. The relaxations of the ^1^G_4_ state populate at the Tm^3+^ ground state (^3^H_6_) emitting at 488 nm and at the ^3^F_4_ state emitting at 650 nm. The population of energy at ^3^H_4_ is a result of non-radiative relaxation from ^3^F_2_. Its relaxation to the ground state emits luminescence at 810 nm in the first biological window. The emission at 1630 nm corresponds to the transition from ^3^F_4_ to the ground state of Tm^3+^ ions involving the single-photon process.

As the ^5^I_6_ level of Ho^3+^ received energy from the ^2^F_5/2_ level of Yb^3+^, ions in the ^5^F_4_/^5^S_2_ states are sequentially excited. The green emission at 548 nm can be obtained after relaxation to the ground state of ^5^I_8_. For the red emission, first the non-radiative relaxation from ^5^I_6_ populates the ^5^I_7_ level, and Ho^3+^ ions continue to receive energy from Yb^3+^ and excite the ^5^F_5_ state, where its relaxation emits red emission at 665 nm. The general mechanism for emission at 1200 nm of Ho^3+^ is a single-photon process, where the energy resident in ^5^I_7_ relaxes to the ground state of ^5^I_8_.

The ^4^I_11/2_ energy level of Er^3+^ is occupied by the energy transferring from the excited Yb^3+^ ions. The higher ^4^I_7/2_ state is excited and non-radiative phonon-assisted relaxations populate the ^2^H_11/2_ and ^4^S_3/2_ levels. The relaxation from these upper levels to the ground state emits green luminescence at 566 nm. The red emission at 660 nm is obtained from the relaxation of the populated ^4^F_9/2_ level to the ground state of ^4^I_15/2_. The excitation energy state ^4^I_11/2_ of Er^3+^ ions coincides with the excited state ^2^F_5/2_ of the Yb^3+^ ions. The excitation light at 980 nm is absorbed mainly by Yb^3+^ and then transfers to the excited state ^4^I_11/2_ of Er^3+^, resulting in a population at ^4^I_13/2_ and an emission at 1530 nm as the relaxation to the ground state of ^4^I_15/2_ takes place.

#### 3.2.2. Upconversion of the Luminescence Spectra

**Figure 3:**
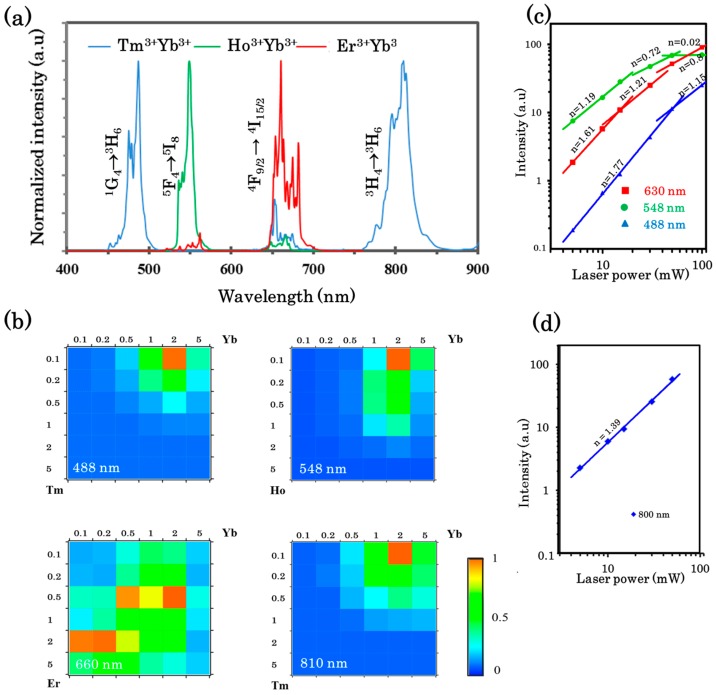
(**a**) Normalized UC emission spectra of Gd_2_O_3_: Tm 0.1 mol %/Yb 2 mol % at 488 nm (blue) and 810 nm (blue), Gd_2_O_3_: Ho 0.1 mol % /Yb 2 mol % at 548 nm (green), and Gd_2_O_3_: Er 0.5 mol %/Yb 2 mol% at 660 nm (red), under 980 nm laser excitation with 10 mW laser power; (**b**) Dopant ratio dependence of the normalized UC emission intensity of Gd_2_O_3_:Tm^3+^/Yb^3+^ at 488 nm and NIR 810 nm, Gd_2_O_3_:Ho^3+^/Yb^3+^ at 548 nm and Gd_2_O_3_:Er^3+^/Yb^3+^ at 660 nm. Double-logarithmic plots of intensity versus excitation power of: (**c**) Gd_2_O_3_: Tm 0.1 mol %/Yb 2 mol % at 488 nm, Gd_2_O_3_: Ho 0.1 mol %/Yb 2 mol % at 548 nm and Gd_2_O_3_: Er 0.5 mol %/Yb 2 mol % at 660 nm, and Gd_2_O_3_: Tm 0.1 mol %/Yb 2 mol % at 810 nm (**d**) under 980 nm laser excitation.

The UC spectra of the Gd_2_O_3_:Ln^3+^/Yb^3+^ (Ln^3+^: Tm^3+^, Ho^3+^, Er^3+^) samples are described in Figure 3a. Gd_2_O_3_ co-doped with Tm^3+^/Yb^3+^ exhibits two dominant peaks at the blue (488 nm) and NIR (810 nm) regions, which are assigned to the 4f-4f transitions of the ^1^G_4_→^3^H_6_ and ^3^H_4_→^3^H_6_ transitions, respectively. The red (650 nm) emission is attributed to the transitions of ^1^G_4_→^3^F_4_. The UC emission spectrum of Gd_2_O_3_ co-doped with Ho^3+^/Yb^3+^ displays a peak at 548 nm (^5^F_4_/^5^S_2_→^5^I_8_) and an emission band at 665 nm (^5^F_5_→^5^I_8_) in their spectra. The UC spectra from Gd_2_O_3_ co-doped with Er^3+^/Yb^3+^ powder consists of two major emission bands around 566 nm and 660 nm, and can be assigned to the transitions of ^2^H_11/2_/^4^S_3/2_→^4^I_15/2_ and ^4^F_9/2_→^4^I_15/2_, respectively.

Figure 3b illustrates the emission intensity at various dopant concentrations as a matrix color scheme. It is well known that the ratio of the dopants contributes greatly to the emission intensity. Herein, the ratio of the activator/sensitizer varies sequentially from 0.1 to 5 mol %. All samples were measured with an incident laser power of 10 mW, and an exposure time of 100 ms. The most favorable ratios determined for three UC emissions in the visible range at 488 nm, 548 nm, and 660 nm were Tm 0.1 mol %/Yb 2 mol %, Ho 0.1 mol %/Yb 2 mol %, and Er 0.5 mol %/Yb 2 mol %, respectively. The value for UC at 810 nm is identical to the emission at 488 nm of Tm^3+^/Yb^3+^.

The dependence of the UC/DC emission intensity and the excitation laser power was investigated by using the samples with the highest intensity and the results are shown in Figure 3c,d. Theoretically, the up/downconverting emission intensity (I_UC/DC_) involves the incident laser intensity (P) as shown in the following equation:

I_UC/DC_ = k P^n^ (3)

where *n* denotes the number of photons required to excite lanthanide ions from the ground state to the emitting excited state, and k is the materials-related coefficient [46]. Practically, the n values are normally smaller than the theoretical integer number because of the competition between the linear decay rate and the upconversion process at the intermediate states [46]. The saturation effect occurs as the decay rate dominates; hence, the saturation significantly decreases the UC efficiency. The generation of higher-energy emissions from lower-energy excitation (UC) is promoted by multiphoton (usually two or three) absorption, resulting in a single emitted photon. In the case of DC, Equation (3) can also apply and a single-photon absorption is assigned to the generation of a lower-energy photon from a higher-energy excitation (DC). A plot of the double logarithm of I_UC_ versus P was fitted by a linear equation having the slope n as shown in Figure 3c,d. The n value for UC emissions is n = 1.77, corresponding to the emissions centered at 488 nm where the three photons are involved in the transition between ^1^G_4_→^3^H_6_ of Tm^3+^/Yb^3+^. Meanwhile, the slopes of the linear fit are 1.19 and 1.61, and the UC emissions at 548 nm and 660 nm depend on the two-photon process, respectively. As seen in Figure 3c, the emissions at 548 nm and 660 nm of NPPs doped with Ho^3+^/Yb^3+^ and Er^3+^/Yb^3+^ saturate at lower irradiance, where the laser power reaches 30 mW. The saturation appears at 50 mW in the emission at 488 nm of Tm^3+^/Yb^3+^, but the slope of 1.39 is unchanged in case of the emission at 810 nm (Figure 3d). The emission at 810 nm of Tm^3+^/Yb^3+^ also involves the two-photon process.

#### 3.2.3. Downconversion of the NIR-Emitting Spectra

**Figure 4:**
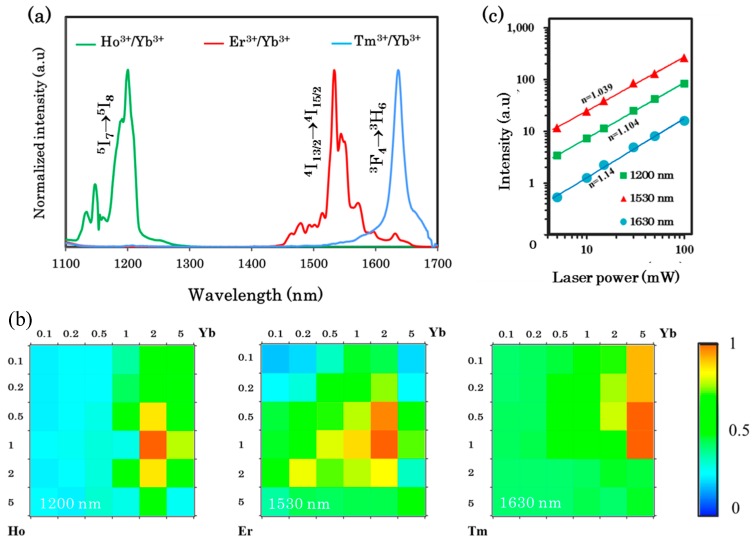
(**a**) Normalized emission spectra in the NIR region of Gd_2_O_3_: Ho 1 mol %/Yb 2 mol % at 1200 nm, Gd_2_O_3_: Er 1 mol %/Yb 2 mol % at 1530 nm, and Gd_2_O_3_: Tm 0.5 mol %/Yb 5 mol% at 1630 nm; (**b**) The dopant composition dependence of the DC emission intensity; (**c**) Dual logarithmic plots of intensity versus excitation power of Gd_2_O_3_: Ho 1 mol %/Yb 2 mol % at 1200 nm, Gd_2_O_3_: Er 1 mol %/Yb 2 mol % at 1530 nm, and Gd_2_O_3_: Tm 0.5 mol %/Yb 5 mol % at 1630 nm, under 980 nm diode laser excitation.

As seen in Figure 4a of the NIR spectra, the NIR emission peaks at 1200 nm, 1530 nm, and 1630 nm are assigned to the presence of Ho^3+^, Er^3+^, and Tm^3+^ ions, respectively. The NIR emission is due to the downconversion process, and the emission peaks can be attributed to the ^5^I_7_→^5^I_8_, ^4^I_13//2_→^4^I_15/2_, and ^3^F_4_→^3^H_6_ transitions, respectively [20,35,47,48,49]. The ratios with the highest intensity obtained for emissions at 1200 nm, 1530 nm, and 1630 nm were Ho 1 mol %/Yb 2 mol %, Er 1 mol %/Yb 2 mol %, and Tm 0.5 mol %/Yb 5 mol %, respectively (see Figure 4). The n-photon mechanism explained in the above can also be applied to elucidating the number of photons involved in the DC process, and the results are exhibited in Figure 4c. The slopes of the straight lines are indicated by n = 1.104, 1.039, and 1.14, corresponding to emissions centered at 1200 nm, 1530 nm, and 1630 nm, respectively. These processes involve one-photon energy transfer [50]. In this work, the saturation has not appeared at up to 100 mW for the three kinds of dopants.

#### 3.2.4. NIR Cellular Imaging

**Figure 5:**
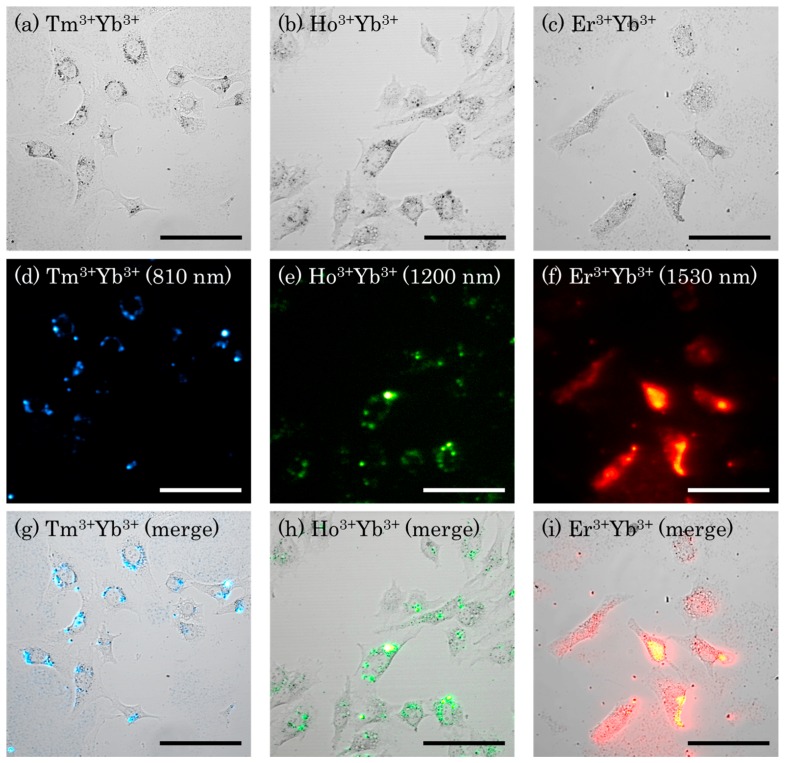
(**a**–**c**) are bright field images of HeLa cell uptake with Gd_2_O_3_: Tm 0.1 mol %/Yb 2 mol % (**a**); Gd_2_O_3_: Ho 1 mol %/Yb 2 mol % (**b**); and Gd_2_O_3_: Er 1 mol %/Yb 2 mol % (**c**); (**d**–**i**) show the emission images in the NIR region of the nanoparticles at 810 nm (**d**), 1200 nm (**e**), 1530 nm (**f**); and their corresponding merged images are shown in (**g**), (**h**), and (**i**), respectively. The scale bars indicate 100 µm.

The dopant ratios with the highest emission intensity at 810 nm (Gd_2_O_3_: Tm 0.1 mol %/Yb 2 mol %), 1200 nm (Gd_2_O_3_: Ho 1 mol %/Yb 2 mol %), and 1530 nm (Gd_2_O_3_: Er 1 mol %/Yb 2 mol %) were applied to produce nanoparticles using the HP method. Figure 5 shows the transmission images of cellular samples incubated with Gd_2_O_3_: Tm 0.1 mol %/Yb 2 mol % (a), Gd_2_O_3_: Ho 1 mol %/Yb 2 mol % (b), and Gd_2_O_3_: Er 1 mol %/Yb 2 mol % (c) and emissions from samples at 810 nm in (d), 1200 nm in (e), and 1530 nm in (f), respectively. The merged images of emissions obtained at 810 nm, 1200 nm, and 1530 nm are shown in Figure 5g–i, respectively. The black spots in the bright field images indicate the distribution of Gd_2_O_3_ doped with Ln^3+^/Yb^3+^ localized in the cells. The emissions of particles localized in the cells were detected at 810 nm, 1200 nm, and 1530 nm, corresponding to the location of the particles in the bright field images. As seen in Figure 5d–f, the NIR emissions from particles uptaken by cells were clearly observed. The merged images show the emission sites are matched with the location of the nanoparticles. At 1530 nm, the elongated luminescence appears. This is attributable to the raster scanning of the incident laser and the long lifetime of the emission of Er^3+^ ions.

In conventional bioimaging, where the UV and visible light are used as an excitation source, the autofluorescence from biological samples interferes with the emissions of dyes and affects the sensitivity of the imaging. With respect to the sample thickness, imaging with UV and visible light is limited in thick samples due to the absorption and scattering of light. The use of NIR light reduces the loss due to scattering and absorption; hence, the light can penetrate into deeper regions. By using Tm^3+^/Yb^3+^ co-doped Y_2_O_3_ incubated with HeLa cells, an emissive image around 810 nm through a 1.5 mm depth of 2% intralipid was reported [32]. The resolution of the image through the 1.5 mm intralipid is comparable to the images without intralipid. This heralds a perspective for microscopic imaging in thick samples such as tissues by employing lanthanide-doped nanophosphors emitting luminescence in the three biological windows.

### 3.3. Cathodoluminescence Images of Nanoparticles

**Figure 6:**
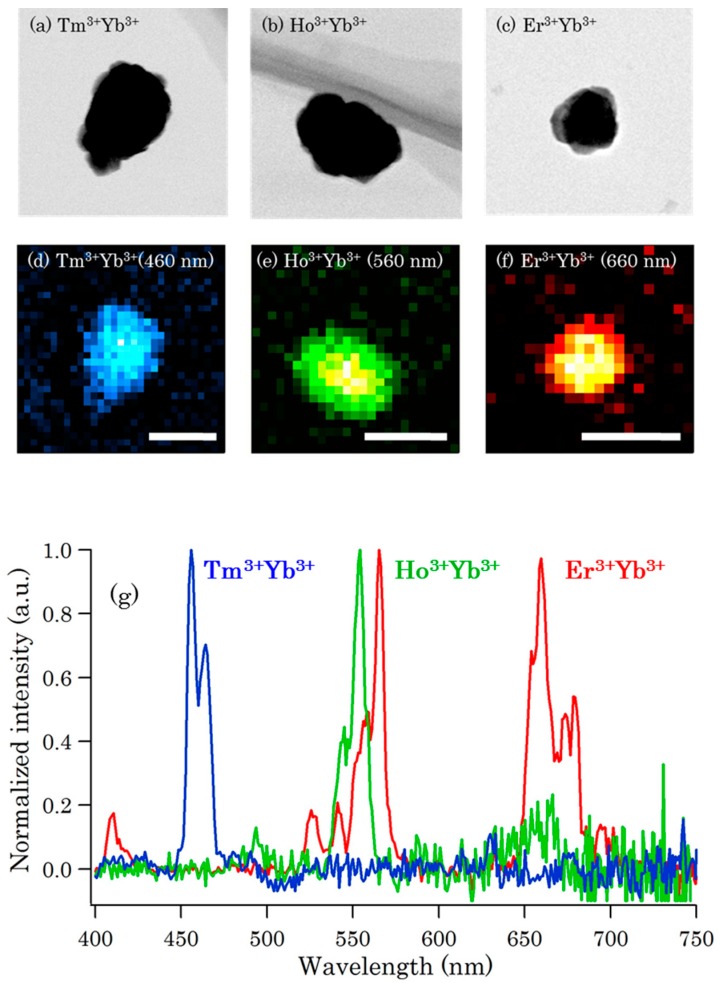
STEM images and CL images of Gd_2_O_3_ co-doped Tm 0.1 mol %/Yb 2 mol % (**a**,**d**); Ho 1 mol %/Yb 2 mol % (**b**,**e**) and Er 1 mol %/Yb 2 mol % (**c**,**f**) nanoparticles; (**g**) is the CL spectra of Gd_2_O_3_ co-doped Tm 0.1 mol %/Yb 2 mol% (blue); Ho 1 mol %/Yb 2 mol % (green) and Er 1 mol %/Yb 2 mol % (red). The scale bars are 100 nm.

The STEM and multi-color CL images of the same nanoparticles as used in NIR cellular imaging (Gd_2_O_3_ co-doped Tm 0.1 mol %/Yb 2 mol %, Ho 1 mol %/Yb 2 mol %, and Er 1 mol %/Yb 2 mol %) under electron beam excitation (accelerating voltage = 80 kV) are shown in Figure 6a–c and Figure 6d–f, respectively. Spectra were taken from aggregates of Gd_2_O_3_ nanoparticles as shown in Figure 6g. Both STEM-CL images and spectra of Gd_2_O_3_ nanoparticles were recorded by using a CL microscope integrated in STEM. Under electron irradiation, both forward and backward emissions from nanoparticles were collected in a parabolic mirror before being focused in front of a spectrometer and EM-CCD.

The group of peaks between 450 and 470 nm of the Gd_2_O_3_ co-doped with Tm^3+^/Yb^3+^ in Figure 6g exhibited the characteristic emissions of Tm^3+^ ions in 4f-4f shell transitions, corresponding to transitions between the levels ^l^D_2_ to ^3^F_4_ the ground state of Tm^3+^ [16]. The transition of ^5^S_2_→^5^I_8_ is assigned to the emission at 540–560 nm for Gd_2_O_3_:Ho^3+^/Yb^3+^ [51]. Regarding Gd_2_O_3_ co-doped Er^3+^/Yb^3+^ (see Figure 6g), an integration of emissions at 650–680 nm is assigned to the transition ^1^F_9/2_→^4^I_15/2_ [52,53] and the group of peaks at 550–575 nm are attributed to the transition of ^4^S_3//2_ and ^2^H_11/2_ to the ground state ^4^I_15/2_ [53,54].

The advantage of electron microscopy is its superior spatial resolution, where conventional optical microscopy is restricted by the Rayleigh criterion:

d = 0.61 λ/NA
(4)

where *d* denotes spatial resolution, *λ* is the wavelength of the light, and the numerical aperture of the objective lens is represented by NA. This diffraction limit hinders the light from resolving the image of two adjacent particles with a size of ~200 nm.

The superior resolution of the electron microscope which uses an electron beam as an alternative optical excitation source can resolve the image of two adjacent particles on the order of the nanometer scale [25]. The advent of the EM and LM combination allows for an object to be fully described at a lower resolution of LM before illustrating its ultrastructure by correlating it to a higher resolution of EM. For exploiting the advantages in the resolution of EM, nanophosphors should have the smallest possible size and a narrow size distribution. In this study, the CL resolution of the image involves a particle diameter of around 100 nm. The CL signals from the particles were clearly detected. As the particle size decreases to 10–30 nm, the localization of the precise position of the biomolecules in the specimen can be achieved. The discrimination of multiple molecules can be visualized by utilizing the multi-color emissions of lanthanide-doped nanophosphors. Multi-color emissions from nanophosphors are attributed to the variation of the dopant elements with their spectral distinction [16,32].

There are several candidate probes which can be useful in immunostaining with CL, such as gold nanoparticles or quantum dots (QDs). Gold nanoparticles and QDs can emit discriminated color under electron beam excitation. However, the CL, due to the plasmon in gold nanoparticles, is relatively weak [55] while the CL of II–VI QDs has relative poor decay [56]. It is also a challenge to obtain the CL from all QDs in the observed area because some QDs do not emit CL. Moreover, both of the probes have size-dependent luminescence. By using multicolor CL emissions from rare earth nanoparticles, the visualization of multiple proteins is possible because color emissions are controlled by activator ions. The CL emission intensity from the Gd_2_O_3_ doped with Ln^3+^/Yb^3+^ nanoparticles was high enough for CL imaging.

In fact, among NIR imaging probes, the sodium fluoride base is the most effective probe. By adding Gd^3+^ ions to its components, an application in MRI is possible [57,58,59]. In order to achieve the correlation of EM and LM, a sodium fluoride base, unfortunately, is inappropriate. Under electron beam irradiation, the sodium fluoride base undergoes a knock-on effect, which results in the collapse of the sodium fluoride base ultrastructure. The application of the sodium fluoride base in CL imaging is consequently hindered [60,61,62]. Particularly in our experience, the cathodoluminescence from oxide matrices doped with Ln^3+^ is superior because they are stable under electron beam bombardment.

## 4. Conclusions

In summary, lanthanide (Tm^3+^, Ho^3+^, Er^3+^, Yb^3+^)-doped Gd_2_O_3_ nanophosphors were prepared by both the sol-gel and HP methods. The NPPs showed a wide range of emissions from visible (blue 488 nm, green 548 nm, red 660 nm) to NIR regions (810 nm, 1200 nm, 1530 nm, and 1630 nm) under 980 nm laser excitation. The variation of emission intensity versus dopant ratios was investigated to obtain the highest emission intensity for each wavelength. The laser power’s dependence on the upconversion emissions shows that the multiphoton process activates the UC emissions. The optimum dopant ratios for emissions at 810 nm (Gd_2_O_3_: Tm 0.1 mol %/Yb 2 mol %), 1200 nm (Gd_2_O_3_: Ho 1 mol %/Yb 2 mol %), and 1530 nm (Gd_2_O_3_: Er 1 mol %/Yb 2 mol %) were applied to produce nanoparticles using the HP method to investigate NIR and CL imaging. The NIR images with HeLa cells under 980 nm excitation and CL images of the nanoparticles under electron beam irradiation were obtained with three kinds of dopants. This result promises potential applications for using Gd_2_O_3_ nanocrystals doped with lanthanides as bioimaging probes, in particular, as dual-modal probes for correlative CL-NIR imaging.

With regards to medical imaging, MRI is one of the most effective imaging techniques to provide the whole-body images. Irrespective of the high-resolution images at an anatomical scale obtained by MRI, the sensitivity of the technique restricts the procurement of detailed information of cell structures and functions in vivo. Because imaging cellular structures and functions requires a higher spatial resolution, which MRI cannot provide, a combination with another high-resolution imaging technique is necessary to provide a high-resolution image of the cellular structures of the target in vivo. The combination of MRI−NIR−CL can potentially allow for the imaging of an object with the scale and spatial resolution spanning from millimeter to nanometer, and a comprehensive image of a biological entity can be obtained. The wide-range scales of CL–NIR–MRI coordination can be used for tracking drug carriers in drug delivery systems (DDS) or for transplanted cell tracking in vivo in regenerative medicine. Cell migration, proliferation, and the condition of transplanted cells are the most important issues in cell tracking systems. The distribution of cells stained by NPPs transplanted in vivo can be tracked by using MRI. The use of high-spatial-resolution techniques such as NIR can provide the single-cell image of the transplanted cells. Different kinds of cells in organs can be discernible by employing three kinds of NPPs emitting at 810 nm, 1200 nm, and 1530 nm. The distribution of particles within the cells (Figure 5) also indicates the potential of observing the cell condition by using microscopic NIR or molecules by using CL.

## Supplementary Materials

The following are available online at www.mdpi.com/2079-4991/6/9/163/s1.
