# Supplementary material for: Multispectral Emissions of Lanthanide-Doped Gadolinium Oxide Nanophosphors for Cathodoluminescence and Near-Infrared Upconversion/Downconversion Imaging
Source: Nanomaterials (Basel). 2016 Sep 6;6(9):163. doi: 10.3390/nano6090163 (PMC5224635; doi:10.3390/nano6090163)
Supplement: Supplementary file 1 [file nanomaterials-06-00163-s001.pdf]

Supplementary Materials: Multispectral Emissions of Lanthanide-Doped Gadolinium Oxide Nanophosphors for Cathodoluminescence and Near-Infrared Upconversion/Downconversion Imaging

Doan Thi Kim Dung, Shoichiro Fukushima, Taichi Furukawa, Hirohiko Niioka, Takumi Sannomiya, Kaori Kobayashi, Hiroshi Yukawa, Yoshinobu Baba, Mamoru Hashimoto and Jun Miyake

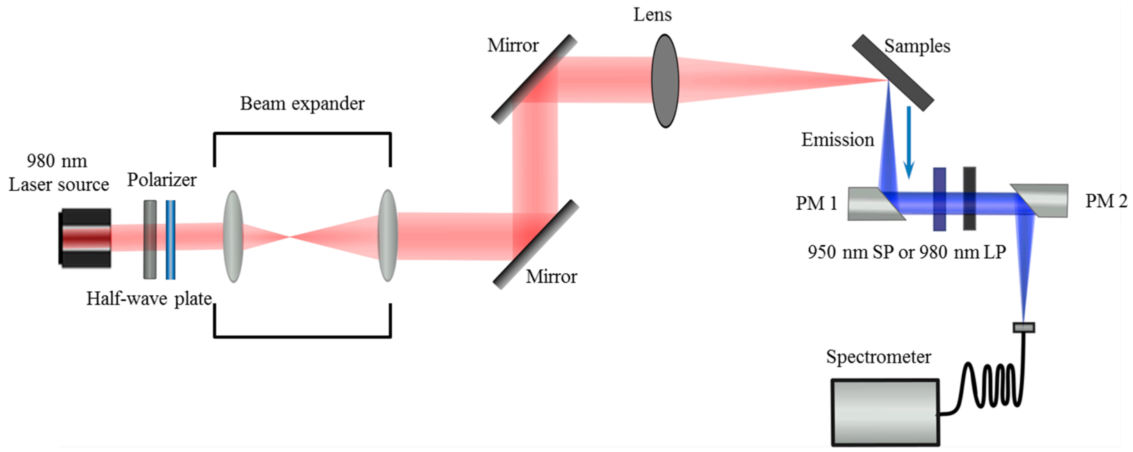

Figure S1. Optical setup to measure UC/DC spectra of Gd_2O_3 co-doped $Tm^{3+}Yb^{3+}$/$Ho^{3+}Yb^{3+}$/ $Er^{3+}Yb^{3+}$. A continuous wavelength (CW) 980 nm diode laser (IRM980TR-500, Laser Century) was set in front of a beam expander. The laser was focused on the sample pellet and the emission from the sample transmits through a silver-coated parabolic mirror (MPD25425-90-P01, Thorlabs; PM 1 and 2) which was used to avoid color aberration, and then was collected to the optical fiber. A 950 nm short-pass filter (FF01-950/SP-25 Semrock) was set in front of PM 2 to cut the reflected laser and the UC emission spectrum was recorded at 298 K by a spectrometer (QE 65 Pro, Ocean Optics). For DC emission measurement, a 980 nm long-pass filter (LP02-980RU-25, Semrock) was inserted to substitute the short-pass filter, and the emission was recorded by a spectrometer (NIRQuest512, Ocean Optics). The spectra obtained were calibrated by using a light source device (HL2000, Ocean Optics.).

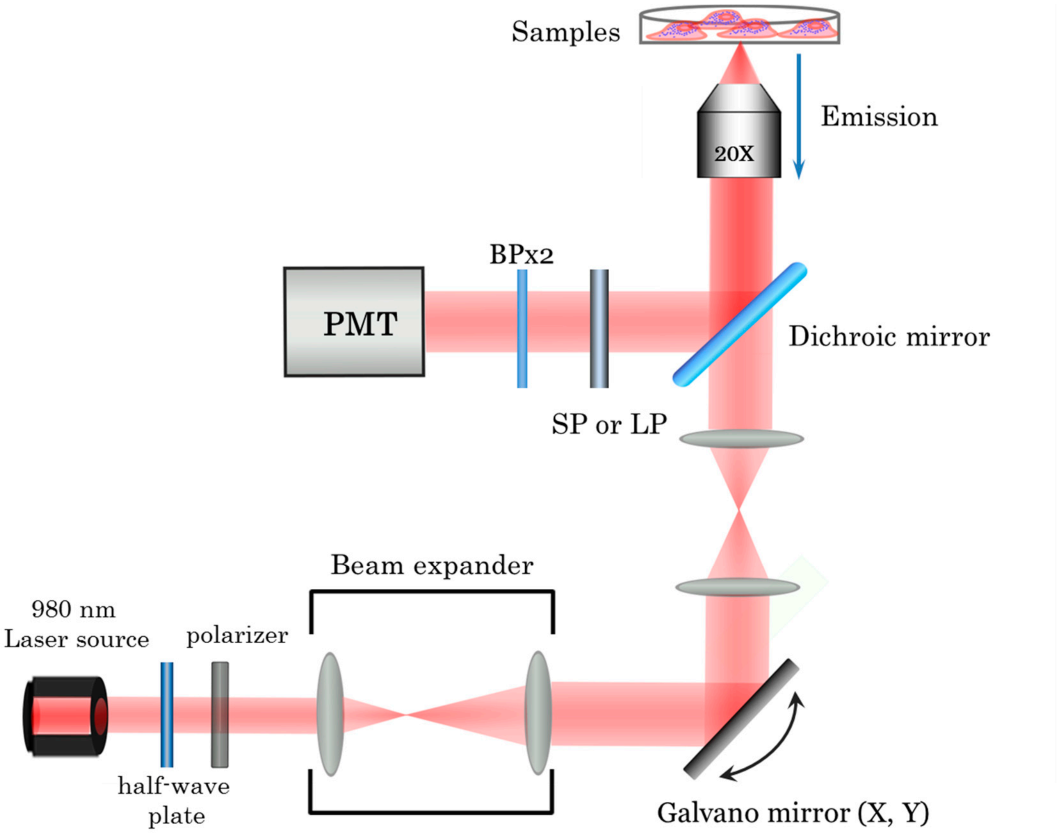

Figure S2. Optical setup for NIR imaging. A 980 nm CW diode laser (IRM980TR–500, Laser Century) was placed and aligned with a beam expander. The laser beam reflected on the galvano mirrors, where its direction was adjusted to reach the dichroic mirror before reaching to the back of the objective lens. The emission was collected to the objective lens and then reflected at the dichroic mirror before being recorded by a photomultiplier tube (PMT). The pixel dwell time was 61.4 microseconds to avoid the elongation of the emission images due to long life time of lanthanide emission.

For emission at 810 nm, the emission from an objective lens (Plan Apo 20X/0.75, DIC M/N2, Nikon) was reflected on a dichroic long–pass filter (FF875–Di01–25x36, Semrock) before being transmitted to a PMT (H7844, Hamamatsu Photonics, Japan). A short-pass filter (FF01–950/SP–25, Semrock) and two bandpass filters (800 nm CWL, 25 mm diameter, Hard Coated, OD 4, 50 nm Bandpass Filter, Edmund Optics) were inserted in front of the PMT to cut the reflected laser and collect the signals at 810 nm.

For emission from an objective lens (Plan Apo NIR 20X/0.40, WD 25, Nikon) at 1200 nm, a 1050 nm dichroic short-pass filter (1050 nm, 25.2 × 35.6 mm, Dichroic Shortpass Filter, Edmund Optics) was used to reflect the emission to the PMT (H10330B-75, Hamamatsu Photonics, Japan). A 980 nm long–pass filter (BLP01-980R-25, Semrock) and two bandpass filters (1200 nm CWL, 25 mm Diameter, Hard Coated, OD 4, 50 nm Bandpass Filter, Edmund Optics) were inserted in front of the PMT to cut the reflected laser and collect the signals at 1200 nm.

In order to obtain the emission from the objective lens (Plan Apo 20X/0.75, DIC M/N2, Nikon) at 1530 nm, a 1200 nm dichroic short-pass filter was replaced (25.2 × 35.6 mm, Dichroic Shortpass Filter, Edmund Optics). The emission was reflected on a dichroic mirror and transmitted through the 980 nm long–pass filter and two bandpass filters (1550 nm CWL, 25 mm Diameter, Hard Coated, OD 4, 50 nm Bandpass Filter, Edmund Optics) before reaching to the PMT (H10330B-75, Hamamatsu Photonics, Japan).

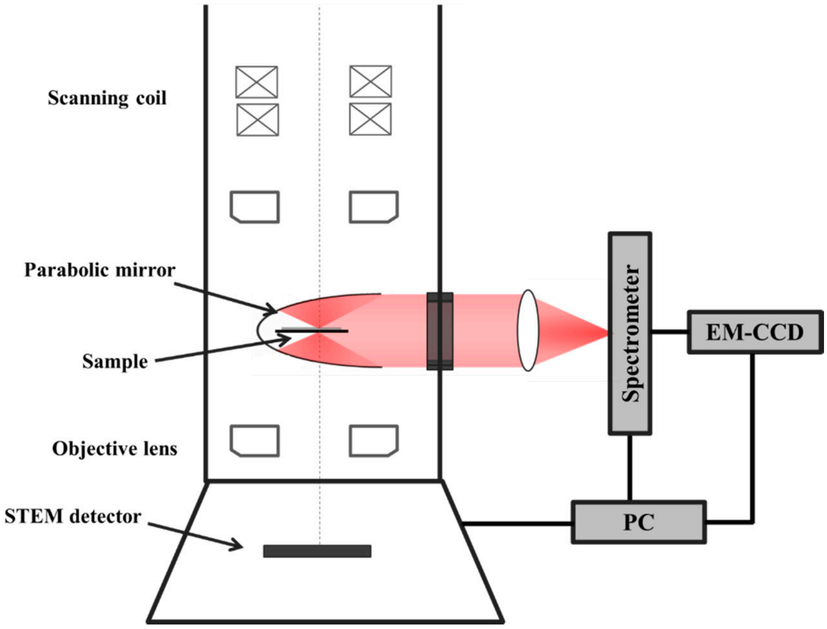

Figure S3. The STEM system (JEM–2100F) integrated with a self-constructed CL system. The electron beam irradiates on the samples and the emitted photons were collected by using an ellipsoidal mirror inserted around the samples to form the parallel rays. The rays escape the STEM system through a quartz window, and focus in front of a slit of the spectrometer (Andor, SR163, *f* = 163 mm, *F*/3.6) by a lens. The CL spectral imaging is detected by an electron multiplying charged–couple device (EM–CCD) detector (Andor, DU920P–BU).

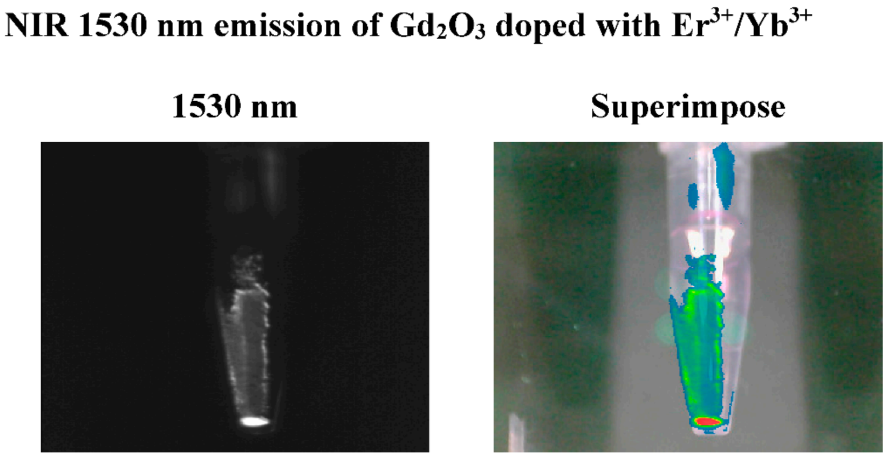

Figure S4. NIR image of Gd_2O_3 co-doped with Er1mol%/Yb2mol% at 1530 nm.

Preparations

Hela cells (3 × 10^5 cells/well) were seeded on a 6-well plate and were incubated for 24 h. The medium (DMEM + 2% FBS) containing-samples were added into the 6-well plate and were labeled overnight. Hela cells were washed by new media, the cells were collected in a microtube, and then the fluorescence was observed by a portable in vivo fluorescence imaging system (SAI-1000; SHIMADZU; excitation laser, 980 nm; emission filter, 1080 nm long-pass).
